# Group A *Streptococcus* NAD-Glycohydrolase Inhibits Caveolin 1-Mediated Internalization Into Human Epithelial Cells

**DOI:** 10.3389/fcimb.2019.00398

**Published:** 2019-11-28

**Authors:** Hirotaka Toh, Ching-Yu Lin, Shintaro Nakajima, Chihiro Aikawa, Takashi Nozawa, Ichiro Nakagawa

**Affiliations:** ^1^Department of Microbiology, Graduate School of Medicine, Kyoto University, Kyoto, Japan; ^2^Department of Life Science Dentistry, The Nippon Dental University, Tokyo, Japan; ^3^Department of Developmental and Regenerative Dentistry, School of Life Dentistry at Tokyo, The Nippon Dental University, Tokyo, Japan

**Keywords:** Group A *Streptoccocus*, internalization, CAV1 (caveolin-1), NAD-glycohydrolase, Streptolysin O (SLO)

## Abstract

Group A *Streptococcus* (GAS) invades epithelial cells causing persistent infection. GAS has a variety of effector proteins that modulate host systems to affect their survival in host environments. The main effector proteins of GAS are NAD-glycohydrolase (Nga) and streptolysin O (SLO). Although Nga has NADase activity and shows SLO-dependent cytotoxicity, some clinical isolates harbor NADase-inactive subtypes of Nga, and the function of NADase-inactive Nga is still unclear. In this study, we found that deletion of *nga* enhanced the internalization of GAS into HeLa and Ca9-22 cells. Amino acid substitution of Nga R289K/G330D (NADase-inactive) does not enhance GAS invasion, suggesting that Nga may inhibit the internalization of GAS into host cells in an NADase-independent manner. Moreover, double deletion of *slo* and *nga* showed similar invasion percentages compared with wild-type GAS, indicating the important role of SLO in the inhibition of GAS invasion by Nga. Furthermore, enhanced internalization of the *nga* deletion mutant was not observed in *Cav1*-knockout HeLa cells. Altogether, these findings demonstrate an unrecognized NADase-independent function of Nga as a negative regulator of CAV1-mediated internalization into epithelial cells.

## Introduction

Group A *Streptococcus* (GAS) or *Streptococcus pyogenes* is an important human pathogen that causes a variety of infections, resulting in a range of symptoms, from mild symptoms such as pharyngitis and impetigo, to severe diseases, such as necrotizing fasciitis and severe invasive streptococcal infection (Walker et al., 2014). GAS can invade and survive in epithelial cells. The intracellular survival of GAS contributes to persistence by escaping from host immune systems and antibiotics, such as penicillin, which shows poor penetration into cells, resulting in asymptomatic infections and invasive diseases (Neeman et al., 1998; Cunningham, 2000).

GAS adheres to and invades epithelial cells *via* endocytotic pathways, particularly *via* cytoskeletal rearrangement using fibronectin-integrin signaling (Molinari et al., 2000; Rohde and Cleary, 2016). GAS harbors a variety of fibronectin-binding proteins, such as streptococcal fibronectin binding protein 1 (Sfb1)/protein F1, protein F2, serum opacity factor, FbaB, glyceraldehyde phosphate dehydrogenase, and several M proteins, which bind to fibronectin in the extracellular matrix of the host (Pancholi and Fischetti, 1992; Natanson et al., 1995; Neeman et al., 1998; Terao et al., 2002; Jeng et al., 2003; Kreikemeyer et al., 2004). Sfb1 and M1 proteins have been shown to induce integrin alpha_5_beta_1_ clustering by binding with fibronectins and activating actin rearrangement through stimulation of phosphatidylinositol 3-kinase and integrin-linked kinase. Sfb1-expressing GAS has also been shown to be internalized from caveolae-like membrane structures (Rohde et al., 2003). Caveolae are flask-shaped regions observed in electroscopic micrographs and in cholesterol- and sphingolipid-rich membranes (Ortegren et al., 2004; Schlormann et al., 2010). Caveolin 1 (CAV1) is a structural protein found in caveolae and is associated with endocytosis of cholera-toxin B subunit and Simian Virus 40 (Montesano et al., 1982; Pelkmans et al., 2001; Shvets et al., 2015). Recently, CAV1 was shown to restrict invasion of GAS into HEp2 cells in a caveolae-independent manner (Lim et al., 2017). However, the mechanisms through which CAV1 regulates invasion, remain unknown.

After GAS invades epithelial cells *via* endocytosis, streptolysin O (SLO) damages the bacterium-containing endosomes and triggers autophagy, a process through which cytosolic GAS cells are targeted by autophagosome-like vacuoles and delivered to lysosomes for degradation (Nakagawa et al., 2004). NAD-glycohydrolase (Nga) is a GAS-secreted protein that catalyzes the hydrolysis of NAD to nicotinamide and adenosine diphosphoribose. Nga is co-transcribed and co-translated with SLO and translocates into epithelial cells in an SLO-dependent manner (Madden et al., 2001; Kimoto et al., 2005; Magassa et al., 2010). Translocated Nga prevents autophagosome maturation and enhances GAS intracellular survival (O'seaghdha and Wessels, 2013), potentially by depleting host NAD and ATP through NADase activity. However, some clinical isolates have been shown to possess an NADase-inactive subtype of Nga (Riddle et al., 2010), which exhibits cytotoxicity in host cells, suggesting that this protein may have NADase-independent functions (Chandrasekaran and Caparon, 2015; Sharma et al., 2016; Hancz et al., 2017). Nga is also involved in the invasion of GAS into keratinocytes (Bricker et al., 2002). However, neither the NADase-independent function of Nga in intracellular GAS nor the molecular mechanisms underlying Nga-regulated GAS internalization have been sufficiently studied.

Accordingly, in this study, we examined the roles of Nga in regulating CAV1-dependent internalization into epithelial cells and showed that Nga was a negative regulator of CAV1-mediated internalization into epithelial cells through an NADase-independent mechanism dependent on SLO.

## Results

### Nga Affected GAS Internalization Into Human Epithelial Cells

**Figure 1:**
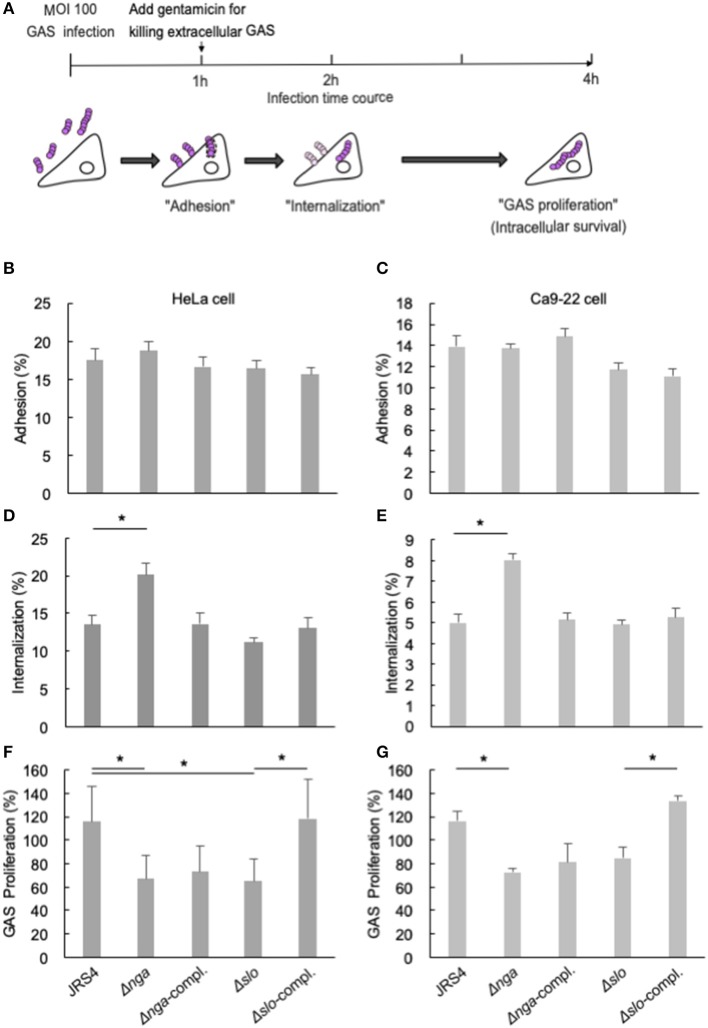
**(A)** Schematic image of gentamicin protection assays. **(B–G)** HeLa cells (wild-type) and Ca9-22 cells were infected with JRS4, Δ*nga* mutant, Δ*nga-*complement, Δ*slo*, and Δ*slo-*complement mutant at a MOI of 100. The percent adhesion (CFU recovered at 1 hpi/infected CFU at 0 h) in **(B)** HeLa cells and **(C)** Ca9-22 cells. The percent internalization (CFU recovered at 2 hpi/CFU at 1 hpi) in **(D)** HeLa cells and **(E)** Ca9-22 cells. The percent GAS proliferation (CFU recovered at 4 hpi/CFU at 2 hpi) in **(F)** HeLa cells and **(G)** Ca9-22 cells. Data represent the means ± SEMs of **(B–G)** five independent experiments. Statistical analysis was performed using pairwise *t*-tests (*p*-values were adjusted using the Bonferroni correction method). Significant differences (*p* < 0.05) are labeled with asterisks.

To evaluate the effects of Nga and SLO during GAS infections in human epithelial cells (Russo et al., 2016), we constructed deletion mutants of the *nga* and *slo* genes using allele exchange methods (Roobthaisong et al., 2017). We infected HeLa cells and Ca9-22 human gingival epithelial cells with these mutants. We then analyzed the adhesion (1/0 h), internalization (2/1 h), and GAS proliferation percentages (4/2 h) of the cells using gentamicin protection assays (Figure 1A). There were no differences in adhesion percentages between wild-type and mutant cells in both cell lines (Figures 1B,C). However, internalization percentages of Δ*nga* mutants were significantly increased compared with that in wild-type GAS (Figures 1D,E) and were rescued by *nga* gene complementation. Additionally, GAS proliferation percentages of Δ*nga* and Δ*slo* mutants were significantly decreased compared with those in JRS4 wild-type cells (Figures 1F,G). Although complementation of the *slo* gene recovered the intracellular proliferation of GAS, the Δ*nga*-complement strain failed to recover (Figures 1F,G).

To explore why complementation of the *nga* gene did not recover intracellular survival, we checked the mRNA level of *nga*, protein expression and secretion of Nga, and NADase activity of bacterial supernatants. Agarose-based reverse transcription polymerase chain reaction (RT-PCR) revealed that *nga* expression in the Δ*nga*-complement strain was comparable to that in the JRS4 wild-type strain (Supplementary Figures 1B,C). However, despite similar levels of Nga in bacterial pellets among JRS4 and Δ*nga*-complement strains, Nga secretion into the culture medium was substantially lower for the Δ*nga*-complement strain than for the JRS4 wild-type strain (Supplementary Figure 1D), implying that the secretion of Nga was defective in the Δ*nga*-complement strain. Similarly, supernatants from the Δ*nga*-complement strain had weaker NADase activity than those from the JRS4 wild-type strain (Supplementary Figure 1E). Given that NADase activity is important for the intracellular survival of GAS in host cells (Sharma et al., 2016), defects in the intracellular proliferation of the Δ*nga*-complement strain may result from quantitative deficiencies in secreted Nga, and NADase activity may not be critical for the inhibitory effects of Nga on internalization.

**Figure 2:**
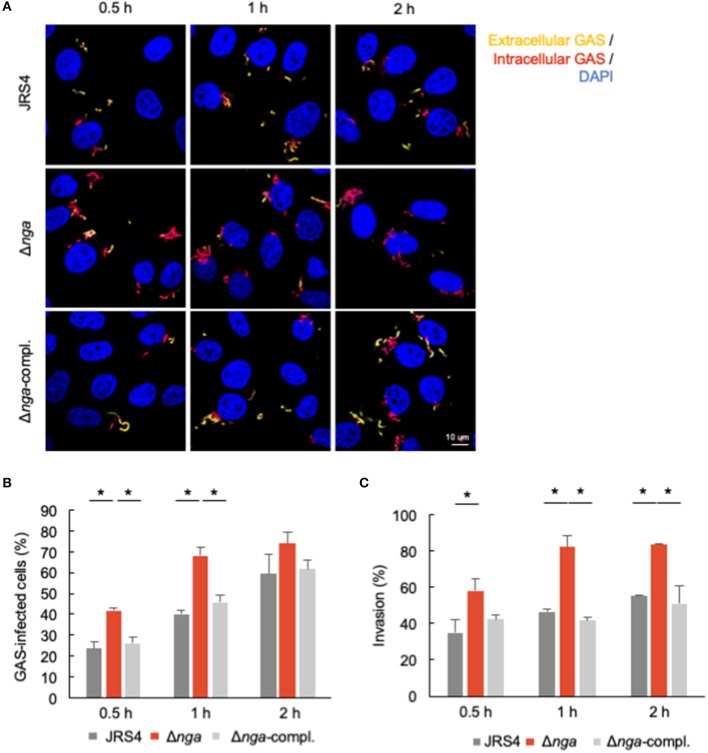
HeLa cells were infected with JRS4, Δ*nga*, and Δ*nga-*complement mutants and differentially immunostained for extracellular and total GAS. Extracellular GAS was labeled with AlexaFluor488, and total GAS was labeled with AlexaFluor594 after Triton-X100 permeabilization. Bacterial/cellular DNAs were stained with DAPI. **(A)** Confocal micrographs of GAS invading into HeLa cells at 0.5, 1, and 2 h. Scale bar: 10 μm. **(B)** Quantification of the percentage of GAS-infected cells. The percentage of GAS-infected cells was determined as AlexaFluor594+ AlexaFluor488– GAS-infected cells divided by the DAPI-visualized cells times 100. More than 100 GAS-infected cells were quantified per independent experiment. **(C)** Quantification of percent invasion. Percent invasion was determined by calculating the number of AlexaFluor594+ AlexaFluor488– GAS divided by the number of AlexaFluor594+ GAS through direct visualization with a confocal microscope. More than 200 AlexaFluor594-positive (total) GAS were quantified per experiment. Data represent the means ± SEMs more than three independent experiments. Statistical analysis was performed by pairwise *t*-tests (*p*-values were adjusted using the Bonferroni correction method). Significant differences (*p* < 0.05) are labeled with asterisks.

To confirm the increased internalization of the *nga* deletion mutant, we investigated the invasion of GAS using differential immunostaining assays. In microscopy experiments, we first determined the percentage of cells with intracellular GAS (red bacteria: intracellular GAS; yellow bacteria: extracellular GAS; Figure 2A). There were significantly more cells with intracellular Δ*nga* GAS than with intracellular JRS4 GAS at the early time points examined (0.5–1 h; Figure 2B); however, at 2 h, we observed only a slight increase in the internalization of Δ*nga* GAS. We next determined the invasion percentages of GAS (intracellular GAS/total GAS). At all-time points (0.5–2 h), we found significant increases in the invasion percentages of Δ*nga* compared with those of JRS4 wild-type and Δ*nga*-complement strains (Figure 2C). Collectively, our results demonstrated that Nga affected GAS internalization into human epithelial cells.

### Nga-Regulated Internalization Was Independent of NADase Activity and Dependent on SLO

**Figure 3:**
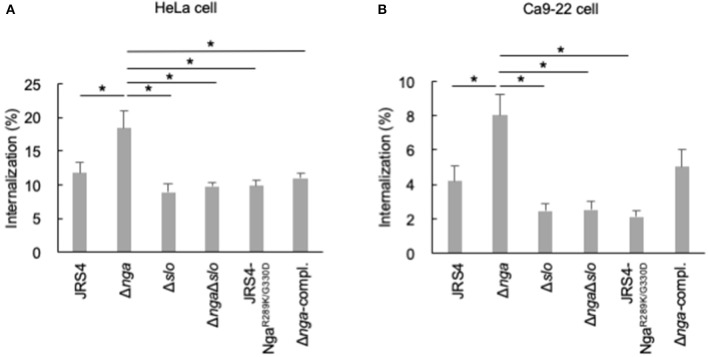
HeLa cells and Ca9-22 cells were infected with JRS4, *nga*, and/or *slo* mutants at a MOI of 100. The percent internalization (CFU recovered at 2 hpi/CFU at 1 hpi) was quantified by gentamicin protection assays **(A)** in HeLa cells and **(B)** Ca9-22 cells. Significant differences (*p* < 0.05) are labeled with asterisks.

Two amino acid residues, i.e., R289 and G330, are located close to the NAD-binding pocket and are required for NADase activity based on the structural analysis of Nga; substitution of these residues abolished NADase activity (Smith et al., 2011; Chandrasekaran et al., 2013). To examine whether Nga-regulated internalization into epithelial cells was dependent on NAD-hydrolysis activity, we generated an endogenous NADase inactive mutant (JRS4-Nga^R289K/G330D^) and infected HeLa and Ca9-22 cells with this mutant. Although the deletion of *nga* significantly enhanced internalization into cells, the NADase-inactive mutant (JRS4-Nga^R289K/G330D^) had an internalization percentage similar to that of the JRS4 wild-type strain (Figures 3A,B), suggesting that Nga-regulated internalization into epithelial cells was an NADase activity-independent process.

Because various reported functions of Nga involve SLO (Michos et al., 2006; Magassa et al., 2010; Bastiat-Sempe et al., 2014), we constructed an *nga*/*slo* double-deletion mutant and examined the invasion efficiency of the mutant. As shown in Figure 3A, enhanced internalization by *nga* deletion was not observed in the *nga*/*slo* double-deletion mutant, suggesting that SLO was involved in Nga-regulated GAS internalization into HeLa cells. Similar results were observed in Ca9-22 cells (Figure 3B). Thus, Nga showed the inhibitory effects on GAS internalization into various epithelial cells through SLO.

### Carbohydrate Recognition Residues of Nga Affected Nga-Regulated Internalization

**Figure 4:**
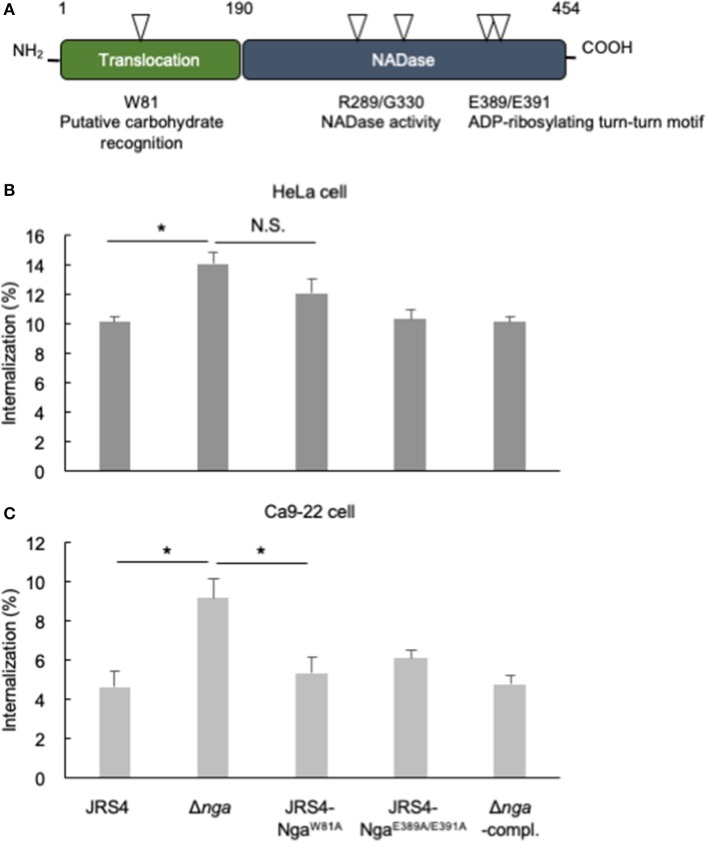
**(A)** The two characteristic domains of Nga. One N-terminal domain was necessary for SLO-dependent translocation, and residue 81 tryptophan was predicted to bind glycan. Another C-terminal domain had a role in NAD-hydrolysis activity; residues arginine 289 and glycine 330 were necessary for NADase activity, and glutamates at positions 389/391 were predicted to be the ARTT-like motif. **(B,C)** HeLa cells and Ca9-22 cells were infected with JRS4, Δ*nga* mutant, Δ*nga-*complement, and JRS4*-*Nga mutants at a MOI of 100. The percent internalization (CFU recovered at 2 hpi/CFU at 1 hpi) was quantified by gentamicin protection assays **(B)** in HeLa cells and **(C)** Ca9-22 cells. Data represent the means ± SEMs of more than three independent experiments. Statistical analysis was performed by pairwise *t*-tests (*p*-values were adjusted using the Bonferroni correction method). Significant differences (*p* < 0.05) are labeled with asterisks.

Tryptophan 81 of Nga is predicted to recognize galactose and is required for translocation to the cytosol as well as for membrane binding of SLO to cholesterol-depleted CHO cells (Mozola and Caparon, 2015). Glutamic acid residues (E389 and E391) of the ADP-ribosylating-turn-turn like loop (ARTT) are homologous to residues of mono ADP-ribosyltransferases, such as the C3 exoenzyme of *Clostridium botulinum* (Ghosh et al., 2010). The C3 exoenzyme transfers an ADP-ribose moiety from NAD to Asn41 of RhoA to enhance binding with RhoGDI and prevents guanine exchange factor activation of Rho, resulting in defective actin polymerization (Aktories et al., 1987; Wiegers et al., 1991; Genth et al., 2003). Although purified Nga from GAS culture supernatants have been reported to show ADP-ribosyltransferase activity (Stevens et al., 2000), purified recombinant Nga expressed in *Escherichia coli* has not been shown to retain ADP-ribosyltransferase activity (Ghosh et al., 2010). To examine whether the sugar recognition and ARTT-like motifs of Nga were associated with internalization of GAS, we generated mutants that have Nga endogenously substituted at W81 and E389/E391 residues with alanine (JRS4-Nga^W81A^ and JRS4-Nga^E389A/E391A^, respectively) by allelic exchange (Figure 4A), and evaluated internalization into HeLa and Ca9-22 cells. The internalization percentage of the JRS4-Nga^W81A^ mutant was similar to that of Δ*nga* in HeLa cells (Figure 4B), suggesting that the sugar recognition motif was involved in mediating the inhibitory effects of Nga on GAS invasion in HeLa cells. In contrast, internalization of the JRS4-Nga^E389A/E391A^ mutant was not increased in HeLa cells (Figure 4B). To confirm these results, we infected Ca9-22 cells with GAS mutants. Interestingly, although Δ*nga* GAS showed enhanced internalization, consistent with the results in HeLa cells, JRS4-Nga^W81A^ did not exhibit increased internalization in Ca9-22 cells (Figure 4C). These findings implied that the glycan profiles of cells may affect the inhibitory functions of Nga on internalization.

Next, we examined whether allelic exchanges in the *nga* gene affected the transcription of other genes, because the *nga* gene consisted of an operon with *sni*, coding the anti-toxin-like protein streptococcal NADase inhibitor, and *slo* (Supplementary Figure 1A; Kimoto et al., 2006). We investigated the mRNA expression of *nga, sni*, and *slo* genes by agarose-based RT-PCR. As shown in Supplementary Figures 1B,C, significant effects on mRNA expression were not observed. Next, to examine whether manipulation influenced protein stability and NADase activity, we evaluated protein levels of Nga and SLO from GAS pellets and culture supernatants by immunoblotting, and measured NADase activity of GAS culture supernatants. As shown in Supplementary Figure 1D, reduced SLO and Nga levels were detected from supernatants of JRS4-Nga^W81A^ or JRS4-Nga^E389A/E391A^ cells compared with that from JRS4 wild-type cells. Consistent with previous reports (Ghosh et al., 2010), supernatants from the JRS4-Nga^E389A/E391A^ strain showed little NADase activity. Additionally, NADase activity in supernatants was decreased in JRS4-Nga^W81A^ cells (Supplementary Figure 1E). Collectively, these results suggested that JRS4-Nga^W81A^ cells may fail to secrete as much Nga protein as JRS4 wild-type cells. However, because the effects of Nga on GAS invasion were rescued by the downregulation of Nga in the Δ*nga*-complement strain, and because similar levels of Nga were detected from the Δ*nga*-complement and JRS4-Nga^W81A^ strains, W81 may be involved in the inhibition of GAS invasion.

### CAV1 Knockout (KO) Rescued the Enhanced Internalization of the Nga Deletion Mutant

**Figure 5:**
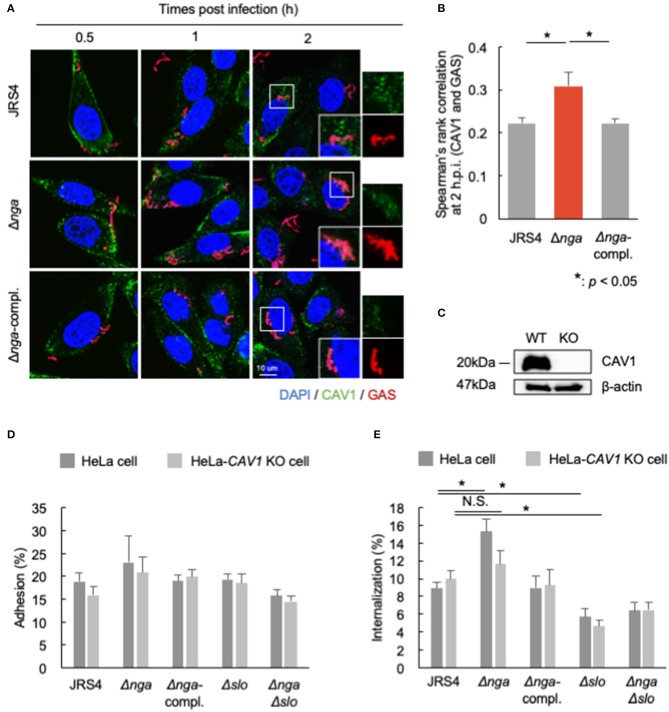
**(A,B)** HeLa cells infected by JRS4, Δ*nga*, and Δ*nga-*complement mutants and immunostained for CAV1 and GAS. **(A)** Confocal micrographs of CAV1 recruitment to GAS at 0.5, 1, and 2 hpi in HeLa cells. Scale bars: 10 μm. **(B)** Quantification of the correlation of CAV1 with GAS at 2 hpi. Spearman's rank correlation coefficients were quantified from more than seven micrograph images per independent experiment using ImageJ/Fiji software. Data represent the means ± SEMs of more than three independent experiments. Statistical analysis was performed by pairwise *t*-tests (*p*-values were adjusted using the Bonferroni correction method). Significant differences (*p* < 0.05) are labeled with asterisks. **(C)** Western blotting of wild-type HeLa cells and HeLa-*CAV1*-KO cells. **(D,E)** HeLa cells and HeLa-*CAV1* KO cells were infected with JRS4, Δ*nga* mutant, Δ*nga-*complement, Δ*slo*, and Δ*nga*Δ*slo* mutant at a MOI of 100. **(D)** Quantification of adhesion percentage and **(E)** the percent internalization using gentamicin protection assays. Data were collected from five independent experiments, and statistical analysis was performed using pairwise *t*-tests (*p*-values were adjusted using the Bonferroni correction method) for wild-type and *CAV1*-KO cells. Significant differences (*p* < 0.05) are labeled with asterisks.

CAV1 directly binds to cholesterol and functions in the formation of intracellular microdomains (Murata et al., 1995). SLO is a cholesterol-dependent cytolysin (Tweten, 2005). In NRK cells, SLO induces the accumulation of caveolae-like vesicles at the plasma membrane and is then internalized into CAV1-positive vesicles for membrane repair (Corrotte et al., 2013). Because Nga-regulated internalization depends on SLO, we suspected that Nga may regulate CAV1 recruitment to the plasma membrane in an SLO-dependent manner. Therefore, we next examined the localization of CAV1 at 0.5, 1, and 2 hpi (Figure 5A). CAV1 was localized in wild-type GAS, the *nga* deletion mutant, and the Δ*nga*-complement mutant; however, these localizations were also partially observed at 0.5 and 1 hpi. Additionally, at 2 hpi, CAV1-localized Δ*nga* GAS was frequently observed (Figure 5A). Spearman's rank correlation between CAV1 and GAS was significantly higher than that with JRS4 and Δ*nga*-complement (Figure 5B), suggesting that Nga inhibited the recruitment of CAV1 to invading GAS.

To test whether CAV1 contributed to the effects of Nga on the inhibition of internalization, we generated *CAV1*-KO cells using the CRISPR/Cas9 system (Figure 5C, Supplementary Figure 2) and examined the adhesion and internalization percentages of Δ*nga* to HeLa-*CAV1*-KO cells. GAS JRS4 and each mutant showed similar adhesion percentages in wild-type HeLa cells and HeLa-*CAV1*-KO cells (Figure 5D). Δ*nga* GAS showed enhanced internalization in wild-type HeLa cells; however, Δ*nga* displayed almost the same level of internalization as wild-type JRS4 in HeLa-*CAV1*-KO cells (Figure 5E). This result suggested that CAV1 may be involved in Nga-regulated internalization into HeLa cells.

## Discussion

**Figure 6:**
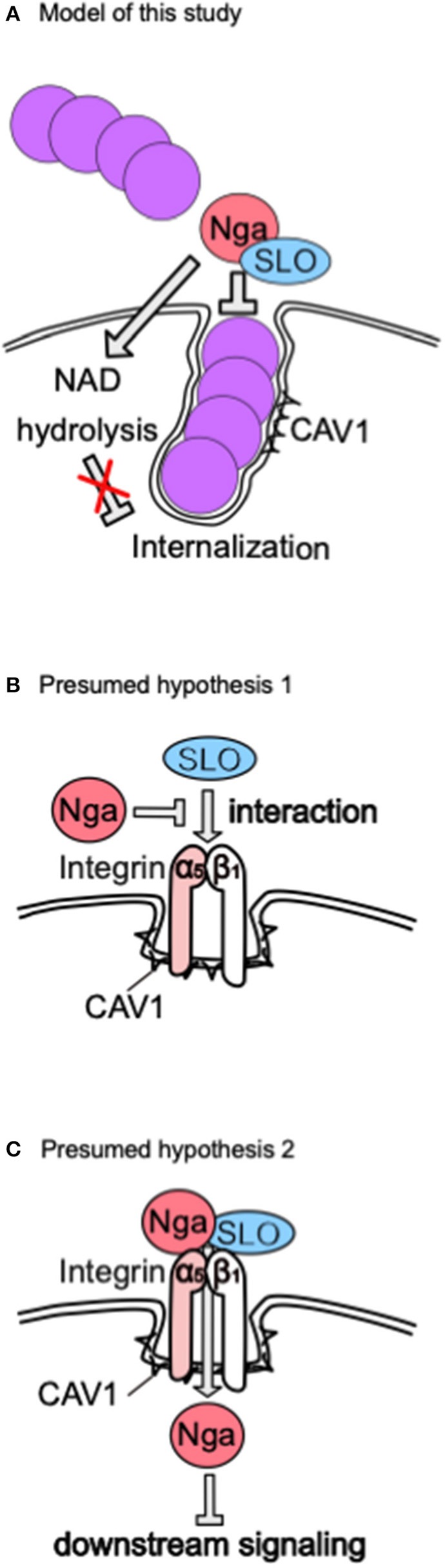
Model of our results and presumed hypothesis. **(A)** Proposal model presumed from our results. **(B)** Hypothesis 1. **(C)** Hypothesis 2. These hypotheses explain how Nga regulates CAV1-mediated internalization in an SLO-dependent manner.

Our results showed that Nga, a protein secreted from GAS, inhibited CAV1-mediated internalization into epithelial cells. This inhibitory function was independent of NADase activity but dependent on SLO (Figure 6A). To the best of our knowledge, this is the first report demonstrating that increased invasion of the Δ*nga* mutant occurred *via* CAV1-mediated internalization.

Nga inhibits uptake into host cells (Bricker et al., 2002), as demonstrated in a study showing that invasion of Δ*nga* strains with background NADase activity was increased. We first demonstrated that NADase activity did not affect Nga-inhibited internalization. NADase-independent cytotoxicity has been reported to activate/accumulate poly-ADP-ribosylation, leading to cell death (Chandrasekaran and Caparon, 2015). Furthermore, this mechanism is known to involve SLO-mediated c-Jun N-terminal kinase (JNK) activation and JNK-dependent cell death (Chandrasekaran and Caparon, 2016). Highly purified Nga does not show biochemical functions, such as ADP-ribosyltransferase and cADPR cyclase activity, but does show NADase activity (Ghosh et al., 2010). Thus, the molecular mechanisms underlying NADase-independent cytotoxicity are still unknown. In this study, we found that the W81A mutant modulated internalization into cells dependent on cell type, consistent with findings predicting that this residue modulates the cytotoxicity of SLO dependent on galactose-containing substances of host cells (Mozola and Caparon, 2015). Notably, SLO recognizes lacto-N-neotetraose with high affinity (Kd = 6.44 × 10^−10^ M) (Shewell et al., 2014). Thus, W81 of Nga may modulate the interactions between Nga and SLO or the SLO receptor on the plasma membrane, depending on the host glycan profile.

Next, we observed the inhibitory effects of SLO on internalization into Ca9-22 cells and HeLa cells. Our results were consistent with Logsdon's report demonstrating that SLO downregulates clathrin-mediated uptake into human keratinocytes (Logsdon et al., 2011). Additionally, previous studies have shown that SLO is endocytosed into CAV1-positive vesicles to modulate membrane repair (Corrotte et al., 2013). CAV1 also colocalizes and regulates integrin alpha_5_beta_1_ endocytosis for fibronectin turnover (Shi and Sottile, 2008). ESCRT induces budding of the wounded plasma membrane by digitonin and perfringolysin O to repair the membrane in HeLa cells (Jimenez et al., 2014). In fibroblasts, ESCRT is involved in the ubiquitination of integrin alpha_5_beta_1_ and degradation of fibronectin-integrin complexes in lysosomes to mediate cell migration (Lobert et al., 2010). Thus, we suspect that differences in membrane repair mechanisms and sensitivity to SLO-induced membrane damage among cell lines may be related to variations in integrin beta_1_ signaling and trafficking.

CAV1 mediates the entry of some bacterial pathogens. In gram-positive bacteria, *Staphylococcus aureus* expresses a fibronectin-binding protein (FnBP) that modulates internalization into epithelial cells *via* the same fibronectin-integrin alpha_5_beta_1_ cascade and actin rearrangement (Sinha et al., 1999; Fowler et al., 2000). Additionally, CAV1 restricts FnBP-dependent uptake of *S. aureus* by limiting membrane microdomain mobility (Hoffmann et al., 2010). In gram-negative bacterial pathogens, CAV1 mediates *Salmonella* uptake by HeLa cells via Rac1 activation and actin reorganization through the effector protein SopE (Lim et al., 2014). Elucidation of the mechanisms through which Nga regulates CAV1-mediated internalization in epithelial cells will provide insights into the roles of CAV1 in bacterial uptake.

In this study, we demonstrated that Nga inhibited internalization *via* an SLO-dependent mechanism. Based on several studies showing that Nga binds SLO (Velarde et al., 2017), Nga translocates into the cytosol in an SLO-dependent manner (Madden et al., 2001; Magassa et al., 2010), *S. aureus* expresses an alpha-hemolysin that interacts with integrin beta_1_ to abolish integrin-dependent adhesion and uptake by A549 epithelial cells (Liang and Ji, 2006), and *S. aureus* and GAS entry into cells occurs *via* integrin alpha_5_beta_1_, we suggest the following alternative hypotheses. First, Nga binds directly to SLO to disrupt the interaction between integrin alpha_5_beta_1_ and SLO or to inhibit integrin activation (Figure 6B). Alternatively, Nga translocates into the cytosol *via* an interaction with the integrin complex and SLO and inhibits integrin downstream signaling (Figure 6C). The integrin complex is a major N-glycan-modified protein, and N-glycan is important for the functions of integrins (Isaji et al., 2009), suggesting that recognition of the host glycan by Nga may modulate the interactions between Nga and SLO or the SLO receptor.

This study had some limitations. First, because we developed this model based on the results of GAS infection with cells, we need to validate this model *via* a biochemical approach, e.g., examining whether Nga inhibits the uptake of Sfb1-labeled beads by epithelial cells with or without SLO. Additionally, because we used JRS4 only as a GAS strain, it is difficult to apply this model to other invasive bacteria expressing the fibronectin-binding proteins M1 and FnBP. The *Streptococcus* cysteine protease SpeB degrades a variety of host and bacterial cell walls and secreted proteins, degrades Nga, and truncates N-terminal SLO (Pinkney et al., 1995; Aziz et al., 2003); SpeB has also been reported to increase the invasion ability of A549 cells (Tsai et al., 1998). Therefore, we speculate that GAS strains showing high expression of SpeB, such as M1T1 GAS, but not low expressions of SpeB, such as JRS4 (Barnett et al., 2013), will exhibit high internalization ability, similar to the JRS4-*nga* deletion mutant, and in contrast to strains with low expression of SpeB, owing to degradation of Nga and production of truncated SLO containing the D4 domain. Therefore, in future works, we will confirm these findings to increase the reliability of this model.

It is still unclear why GAS restricts its internalization into host epithelial cells. Although GAS can invade into host cells, intracellular pathways, such as the endosome-lysosome pathway and autophagy, degrade invading GAS. Given that GAS can translocate Nga into the host cytosol *via* SLO-dependent mechanisms without invading cells to modulate host signaling (Magassa et al., 2010; Hancz et al., 2017), there may be multiple advantages to avoid invading host cells by GAS. In particular, because NADase activity is critical for intracellular survival of GAS (Bastiat-Sempe et al., 2014), NADase-inactive GAS may show limited internalization into host cells. Overall, our findings provides evidence that Nga has inhibitory effects on GAS internalization into human epithelial cells in an NADase activity-independent and SLO-dependent manner.

## Materials and Methods

### Bacterial Strains

Group A *Streptococcus* strain JRS4 (M6^+^, F1^+^) and gene deletion mutants were grown in Todd-Hewitt broth (BD Diagnostic Systems) supplemented with 0.2% yeast extract (THY) as previously described (Nakagawa et al., 2004) and Tryptic-Soy broth supplemented with 0.2% yeast extract for bacterial adhesion, internalization, and GAS proliferation assays. The GAS strains used in this study are listed in Supplementary Table 1.

### Cell Lines, Culture Conditions, and Transfection

HeLa cells and Ca9-22 cells were maintained in Dulbecco's modified Eagle's medium (DMEM; Nacalai Tesque) supplemented with 10% fetal bovine serum (JRH Biosciences) and 50 μg/ml gentamicin (Nacalai Tesque) in a 5% CO_2_ incubator at 37°C. The transfection reagents used were polyethylenimine (Polysciences) and Lipofectamine 3000 (Invitrogen, Carlsbad, CA, USA).

### Antibodies and Reagents

For western blotting, anti-beta-actin (1:1,000; cat. no. D6A8; Cell Signaling Technology, Danvers, MA, USA), anti-CAV1 (1:1,000; cat. no. D46G3; Cell Signaling Technology), anti-SLO (1:1,000; Cosmo Bio), and anti-Nga (1:1,000; cat. no. 64-005; Bio Academia) antibodies were used as primary antibodies, and horseradish peroxidase-conjugated anti-mouse IgG and anti-rabbit IgG antibodies (1:5,000; Jackson ImmunoResearch Laboratories) were used as secondary antibodies. For immunostaining experiments, anti-CAV1 (1:400; cat. no. D46G3; Cell Signaling Technology) and anti-GAS (1:250 or 1:500; cat. no. PAB13831; Abnova and cat. no. ab9191; Abcam) antibodies were used as primary antibodies, and anti-mouse, anti-rabbit, or anti-goat IgG conjugated with AlexaFluor-488 and AlexaFluor-568 (1:250; Molecular Probes/Invitrogen) or AlexaFluor-594 (1:250; Jackson ImmunoResearch Laboratories) antibodies were used as secondary antibodies. 4′,6-Diamidino-2-phenylindole (DAPI; 1:1,000; Dojindo) was used to stain bacterial and cellular DNA.

### Generation of Gene-Deletion, Complemented, and Amino Acid-Substituted Mutants

A two-step allele exchange by the thermo-sensitive vector pSET4s (Takamatsu et al., 2001) was used to delete each gene, as described previously (Roobthaisong et al., 2017). A schematic procedure of genotype manipulation is shown in Supplementary Figure 3. Briefly, to construct the pSET4s vector carrying each gene-deletion allele, 5′ and 3′ flanking regions (each 800 bp) of each gene were amplified from wild-type genomic DNA, joined to *Sma*I-digested pSET4s by Gibson Assembly (New England BioLabs), and transformed into *E. coli* DH10B. Clones selected on 100 μg/ml spectinomycin (Nacalai Tesque) in LB agar plates were confirmed to contain the deletion allele by colony PCR. Purified plasmids were electro transformed into wild-type JRS4 cells and plated on 100 μg/ml spectinomycin in THY agar plates at a permissive temperature of 28°C. To generate chromosomal single-crossover mutants, selected colonies were grown at a non-permissive temperature of 37°C with spectinomycin. To induce the second crossover event, single-crossover mutants confirmed to exchange a chromosomal allele were then subcultured to a permissive temperature of 28°C without spectinomycin. Spectinomycin-sensitive colonies were screened for either gene deletion or returned to the wild-type genotype by colony PCR. To complement the *nga* gene in the Δ*nga* mutant, the 5′ to 3′ flanking region containing the *nga* gene (1,600 + 1,356 bp) was amplified from wild-type genomic DNA and joined with *Sma*I-digested pSET4s. pSET4s carrying the complement gene was then electrotransformed into the Δ*nga* mutant, and the deletion method described above was carried out. To substitute amino acids in Nga, the *nga* and *sni* genes were amplified and joined to pSET4s. Target regions were substituted by inverse PCR. Plasmids were electrotransformed into JRS4, and the deletion method was then performed. Target substitutions were sequenced to confirm the desired mutations. Primers are listed in Supplementary Table 2.

### Bacterial Adhesion, Internalization, and GAS Proliferation Assays

HeLa or Ca9-22 cells were seeded in 24-well plates (Nunc) at 5 × 10^4^ cells/well and infected with GAS at a multiplicity of infection (MOI) of 100. After an appropriate incubation period, cells were washed with phosphate-buffered saline (PBS), treated with 100 μg/ml gentamicin in DMEM to kill extracellular bacteria, and incubated for evaluation of internalization and intracellular GAS proliferation efficiency. At each time point, cells were lysed with sterile Milli-Q water, and lysates were plated on TSA agar plates to count colony forming units (CFU). Adhesion efficiency was calculated as the ratio of the number of recovered cells at 1 h to the number of suspended cells at 0 h. Internalization efficiency was calculated as the ratio of the number of recovered cells at 2 h to the number of recovered cells at 1 h. GAS proliferation efficiency was calculated as the ratio of the number of recovered cells at 4 h to the number of recovered cells at 2 h.

### Microscopic Bacterial Internalization Assay

Epithelial cells (HeLa or Ca9-22 cells) were seeded onto coverslips (Matsunami Glass) coated with 0.1% gelatin (BD Diagnostic Systems) in 24-well plates at 5 × 10^4^ cells/well, followed by infection with GAS at a MOI of 100. Cells were fixed for 20 min with 4% paraformaldehyde in PBS and washed with PBS. Extracellular bacteria were then stained with anti-GAS (1:500) and anti-rabbit IgG AlexaFluor488 (1:500) antibodies at room temperature for 1 h, permeabilized with 0.1% Triton in PBS for 15 min, washed with PBS, and blocked at room temperature for 1 h with 2% bovine serum albumin (BSA; cat. no. A4053; Sigma-Aldrich, St. Louis, MO, USA) and 0.02% NaN_3_ in PBS. Intracellular and extracellular bacteria were stained with anti-GAS antibodies (1:250) at 4°C overnight and anti-rabbit IgG AlexaFluor594 (1:250) at room temperature for 2–3 h. To visualize bacterial and cellular DNAs, samples were stained with DAPI. Confocal fluorescence micrographs were acquired with an FV1000 laser-scanning microscope (Olympus). GAS-infected cells (%) represent the number of invading GAS-positive cells divided by the number of DAPI-positive cells times 100. The invasion rate was calculated as the number of intracellular GAS (AlexaFluor594+/AlexaFluor488–) divided by the number of total GAS (AlexaFluor594+) times 100.

### Generation of KO Cell Lines by CRISPR/Cas9

CRISPR/Cas9 was used to generate HeLa-CAV1-KO cells, as described previously (Oda et al., 2016). Briefly, a CRISPR guide RNA targeting the first exon of *CAV1* was designed (5′-GGCAAATACGTAGACTCGG-3′) and ligated with the gRNA-hyg vector. HeLa cells were transfected with the hCAS9 vector (Neo^R^; Addgene 41815) and the *CAV1*-targeted gRNA-hyg vector. Cells transfected with both vectors were selected with 300 μg/ml hygromycin B (Nacalai Tesque) and 750 μg/ml geneticin (G418; Nacalai Tesque). Single colonies were expanded, and KO was confirmed by western blotting. Second, genomic DNA was isolated from cells, and target regions were amplified by PCR. Target regions were sequenced to confirm the presence of frameshift insertions or deletions.

### Nga and SLO Immunoblotting of Bacterial Pellets and Culture Supernatants

Overnight cultures were inoculated into fresh culture medium and grown to late-exponential phase (OD_600_ of 0.8). Bacterial cultures were centrifuged at 11,000 × *g* for 3 min at room temperature, and supernatants were passed through a 0.2-μm filter (Corning, USA). GAS pellets or supernatants were mixed with 2 × sodium dodecyl sulfate polyacrylamide gel electrophoresis (SDS-PAGE) sample buffer, boiled at 100°C for 10 min, separated by SDS-PAGE, and transferred to polyvinylidene difluoride membranes (0.45 μm; Merck Millipore). Nga and SLO bands were visualized by standard immunoblotting and chemiluminescence methods.

### NADase Activity Assay of GAS Culture Supernatants

NADase activity in GAS culture supernatants was determined by measuring the fluorescence intensity as described by Madden et al. (2001). Briefly, 200 μl of each supernatant was two-fold serial diluted by PBS until reaching 256-fold dilution, mixed with 50 μl of 1 mM β-NAD (Nacalai Tesque), and incubated at 37°C in a 5% CO_2_ incubator for 1 h. The reactions were stopped by the addition of 100 μl of 5 M NaOH (Nacalai Tesque), and samples were incubated at room temperature for 1 h. The concentration of NAD+ was determined by measuring the intensity (460 nm emission upon 340 nm excitation) with a plate reader (Wallac ARVO SX Multilabel Counter; Perkin Elmer, Waltham, MA, USA) compared with known concentrations of NAD+. NADase activity was calculated as nmol NAD cleaved/min and normalized to that in the JRS4 wild-type strain.

### Comparison of Gene Expression by Agarose Electrophoresis-Based RT-PCR

RNA extraction from GAS was performed as described by Chiang-Ni et al. (2009). Briefly, GAS collected from late-log phase (OD_600_ of 0.8) were resuspended with RNA Protect Bacteria Reagent (Qiagen, Valencia, CA, USA) to stabilize the bacterial RNA for 10 min at room temperature, resuspended with lysis buffer (10 mM Tris-HCl [pH 8.0], 2 mM ethylenediaminetetraacetic acid, 20 mg/ml lysozyme [Nacalai Tesque], and 100 U/ml mutanolysin [Sigma]), and stored at −80°C overnight. Following thawing at room temperature, samples were incubated at 37°C for 1 h. Total RNA was isolated by NucleoSpin RNA (Macherey Nagel) with on-column DNase digestion and reverse-transcribed to cDNA using PrimeScript II 1st strand cDNA Synthesis (Takara Bio, Shiga, Japan). Genes were amplified with *TaKaRa ExTaq*-HS (Takara Bio) and specific primers described by Hsieh et al. (2018) using the following steps: 50°C for 5 min; 95°C for 2 min; 30 cycles of 95°C for 5 s and 62°C for 30 s; and 62°C for 1 min. Amplified PCR products were separated by 2% agarose-gel electrophoresis, stained with ethidium bromide, and visualized with UV emission. For comparison of gene expression between samples, band intensities were quantified using ImageJ/Fiji software. Gene expression was calculated as a ratio to *gyrA*, the internal control, and shown as the value relative to that in the JRS4 wild-type strain. PCR product sizes were as follows: *nga*, 182 bp; *slo*, 169 bp; *sni*, 173 bp; *gyrA*, 144 bp.

### Fluorescence Microscopy

Cells were seeded onto 0.1% gelatin-coated coverslips in 24-well plates at 1 × 10^5^ cells/well and then infected with GAS at a MOI of 100. Cells were fixed for 15 min with 4% paraformaldehyde in PBS, washed with PBS, permeabilized with 0.1% Triton X-100 in PBS for 10 min, and blocked by 2% BSA and 0.02% NaN_3_ in PBS at room temperature for 1 h. Cells were then probed with primary antibodies in blocking solution at 4°C overnight and labeled with secondary antibodies at room temperature for 2 h. Cellular and bacterial DNAs were stained with DAPI. Confocal fluorescent micrographs were acquired with an FV1000 laser-scanning microscope.

### Statistical Analysis

Unless otherwise indicated, values included in graphs represent the mean ± standard errors of the means (SEMs) calculated from more than three independent (biological) replicates. Statistical analysis was performed by two-tailed Student's *t*-tests for two groups or by pairwise *t-*tests (the *p*-value was adjusted by Bonferroni's correction) for more than two groups. Results with *p*-values of <0.05 were considered to indicate statistical significance.

## Data Availability Statement

The raw data supporting the conclusions of this manuscript will be made available by the authors, without undue reservation, to any qualified researcher.

## Author Contributions

HT performed the experiments, analyzed the data, and drafted the manuscript. SN and C-YL assisted with data collection. TN, CA, and IN conceived the study, provided reagents, and revised the paper.

### Conflict of Interest

The authors declare that the research was conducted in the absence of any commercial or financial relationships that could be construed as a potential conflict of interest.

## Supplementary Material

Supplementary Table 1 GAS strains used in this study.

Supplementary Table 2 Primers used to delete the *nga* and *slo* genes, generate a chromosomal-complement *nga* gene for the *nga* deletion mutant, mutate amino acids in Nga, and amplify genes from cDNA using agarose-based RT-PCR.

Supplementary Figure 1 **(A)** Schematic illustration of the *nga*-*sni*-*slo* operon. Characters shown as “P” and “T” represent promoters and terminators, respectively. **(B)** Representative images of agarose gel electrophoresis of amplified PCR products for each gene (*nga, slo, sni*, and *gyrA*). **(C)** Relative gene expression of *nga, slo*, and *sni* among GAS mutants. Band intensities in agarose electrophoresis images were quantified using ImageJ/Fiji, calculated as a ratio to *gyrA*, and reported as values relative to those in the JRS4 wild-type strain. **(D)** Representative images of western blotting of Nga and SLO in pellets and supernatants (OD_600_ of 0.8). Immunoblotting images were taken at short (8 s) and long (30 s) exposure times. **(E)** Relative NADase activity in GAS mutants. NADase activities in GAS mutants were determined from culture supernatants at the late-exponential phase (OD_600_ of 0.8) and shown as values relative to those in the JRS4 strain. Data represent the means ± SEMs of four independent experiments.

Supplementary Figure 2 Sequence results of genomic *CAV1* regions targeted by the gRNA of wild-type and *CAV1*-KO HeLa cells. Red letters represent the deleted regions, bold letters indicate the initiation codon, underlining shows the gRNA target site, and blue letters represent the PAM motif.

Supplementary Figure 3 Schematic procedure of allelic exchange for **(A)** chromosomal gene deletion, **(B)** chromosomal gene complementation, and **(C)** amino acid substitution in nga.
