# Supplementary material for: Group A Streptococcus NAD-Glycohydrolase Inhibits Caveolin 1-Mediated Internalization Into Human Epithelial Cells
Source: Front Cell Infect Microbiol. 2019 Nov 28;9:398. doi: 10.3389/fcimb.2019.00398 (PMC6893971; doi:10.3389/fcimb.2019.00398)
Supplement: Supplementary Table 1 — GAS strains used in this study. [file Table_1.pdf]

Supplementary table 1. GAS strains used in this study

Strains	Genotype/characteristics	Reference
JRS4	Wild type, $M6^{+}F1^{+}$, a streptomycin-resistant derivative of a clinical isolate	Nakagawa I et al., 2004
Δ*nga*	*nga* deletion mutant of JRS4	This study
Δ*slo*	*slo* deletion mutant of JRS4	This study
Δ*nga*Δ*slo*	Double (*nga* and *slo*) gene deletion mutant of JRS4	This study
Δ*nga*-complement	Genomic *nga* gene-complemented mutant of Δ*nga*	This study
Δ*slo*-complement	Genomic *slo* gene-complemented mutant of Δ*slo*	This study
JRS4-$Nga^{R289K/G330D}$	Genomic nga mutant of JRS4, Lys substitution at Arg289 and Asp substitution at Gly330, catalytically inactive	This study
JRS4-Nga^{W81A}	Genomic nga mutant of JRS4, Ala substitution at Trp81, putative carbohydrate binding residue	This study
JRS4-$Nga^{E389A/E391A}$	Genomic nga mutant of JRS4, Ala substitution at Glu389 and Glu391, an ADP-ribosylating turn-turn motif	This study
