# Supplementary material for: Group A Streptococcus NAD-Glycohydrolase Inhibits Caveolin 1-Mediated Internalization Into Human Epithelial Cells
Source: Front Cell Infect Microbiol. 2019 Nov 28;9:398. doi: 10.3389/fcimb.2019.00398 (PMC6893971; doi:10.3389/fcimb.2019.00398)
Supplement: Supplementary Table 2 — Primers used to delete the nga and slo genes, generate a chromosomal-complement nga gene for the nga deletion mutant, mutate amino acids in Nga, and amplify genes from cDNA using agarose-based RT-PCR. [file Table_2.pdf]

Supplementary table 2. Primer for gene-deletion, complementation, amino-acid substitution

Primer name	Sequence (5' – 3')	Application
spy0146upper800_fwd	tcgagctcggtaccccCAATATTTTGCGCGTCGAAATTC	*nga* deletion, complementation
spy0146upper800_rev	atacatattgGTAAACCACCTTATATTATTTTTAAGTAAAC	*nga* deletion
spy0146down800_fwd	gtggtttacCAATATGTATAAGGTGCCAAAG	*nga* deletion
spy0146down800_rev	ctctagaggatccccTCTGACTTTTTTTCTTCTTTTTCTG	*nga* deletion, complementation
spy0148upper800_fwd	tcgagctcggtaccccATAAGCTAGGCTATATCCGAAC	*slo* deletion, complementation
spy0148upper800_rev	aaccagtcACCTTTTTATCATTCTAAAATGTTTC	*slo* deletion
spy0148down800_fwd	ataaaaaggtGACTGGTTCAAGAGGTTCGTC	*slo* deletion
spy0148down800_rev	ctctagaggatccccACTTTACACTGTATGGTAAGCC	*slo* deletion, complementation
pSET4S_nga/sni_fwd	tcgagctcggtacccatgagaaacaaaaaagtaacattag	Nga mutants construct
pSET4S_nga/sni_rev	ctctagaggatccccctaaaatgtttctattgttctttcga	Nga mutants construct
nga R289K fwd	gctaaaaaaaagtcaacgattggtcgacaagaa	Inverse PCR
nga R289K rev	tgactttttttagcatcatcaaaagctaacat	Inverse PCR
nga G330D fwd	ataaaagatgtcgatagcggaaaatatagtgat	Inverse PCR
nga G330D rev	atcgacatcttttatattttcaatttggtcagc	Inverse PCR
nga W81A fwd	actgtcgcggaggaaaattcacctggtg	Inverse PCR
nga W81A rev	ttcctccgcgacagtactcattgagccg	Inverse PCR
nga E389A/E391A fwd	aattcagcaagtgcattaattttcccatcgattagtgtt	Inverse PCR
nga E389A/E391A rev	aattaatgcacttgctgaattttcaatgtgttttcctga	Inverse PCR
nga RT-PCR fwd	TGTTGCTATTGCTTTGGCTG	RT-PCR
nga RT-PCR rev	TTGAGCCGTCTAATGTGTGC	RT-PCR
slo RT-PCR fwd	TCCTGCGGATGTGTTTGATA	RT-PCR
slo RT-PCR rev	TGCACTAAAGGCCGCTTC	RT-PCR
sni RT-PCR fwd	AGAAATGTCAAATAGCGGTCAAG	RT-PCR
sni RT-PCR rev	CCATAGCCTCTCTAATATGCGC	RT-PCR
gyrA RT-PCR fwd	CGTCGTTTGACTGGTTTGG	RT-PCR
gyrA RT-PCR rev	GGCGTGGGTTAGCGTATTTA	RT-PCR
