# Supplementary figures and images for: Group A Streptococcus NAD-Glycohydrolase Inhibits Caveolin 1-Mediated Internalization Into Human Epithelial Cells
Source: Front Cell Infect Microbiol. 2019 Nov 28;9:398. doi: 10.3389/fcimb.2019.00398 (PMC6893971; doi:10.3389/fcimb.2019.00398)

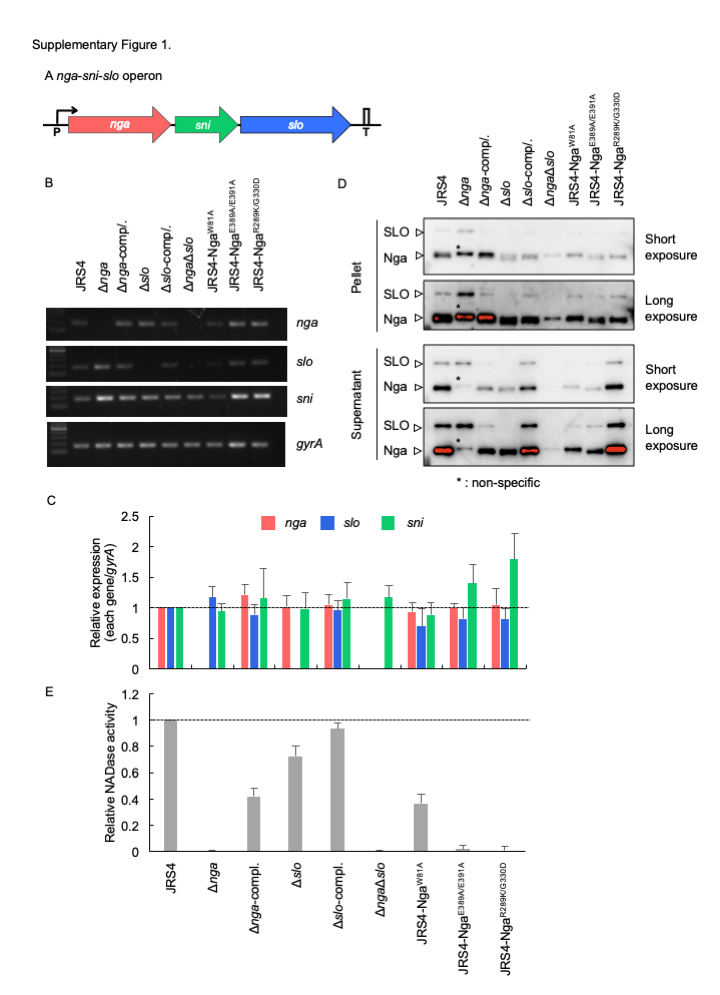

Supplement: Supplementary Figure 1 — (A) Schematic illustration of the nga-sni-slo operon. Characters shown as “P” and “T” represent promoters and terminators, respectively. (B) Representative images of agarose gel electrophoresis of amplified PCR products for each gene (nga, slo, sni, and gyrA). (C) Relative gene expression of nga, slo, and sni among GAS mutants. Band intensities in agarose electrophoresis images were quantified using ImageJ/Fiji, calculated as a ratio to gyrA, and reported as values relative to those in the JRS4 wild-type strain. (D) Representative images of western blotting of Nga and SLO in pellets and supernatants (OD600 of 0.8). Immunoblotting images were taken at short (8 s) and long (30 s) exposure times. (E) Relative NADase activity in GAS mutants. NADase activities in GAS mutants were determined from culture supernatants at the late-exponential phase (OD600 of 0.8) and shown as values relative to those in the JRS4 strain. Data represent the means ± SEMs of four independent experiments. [file Image_1.tiff]

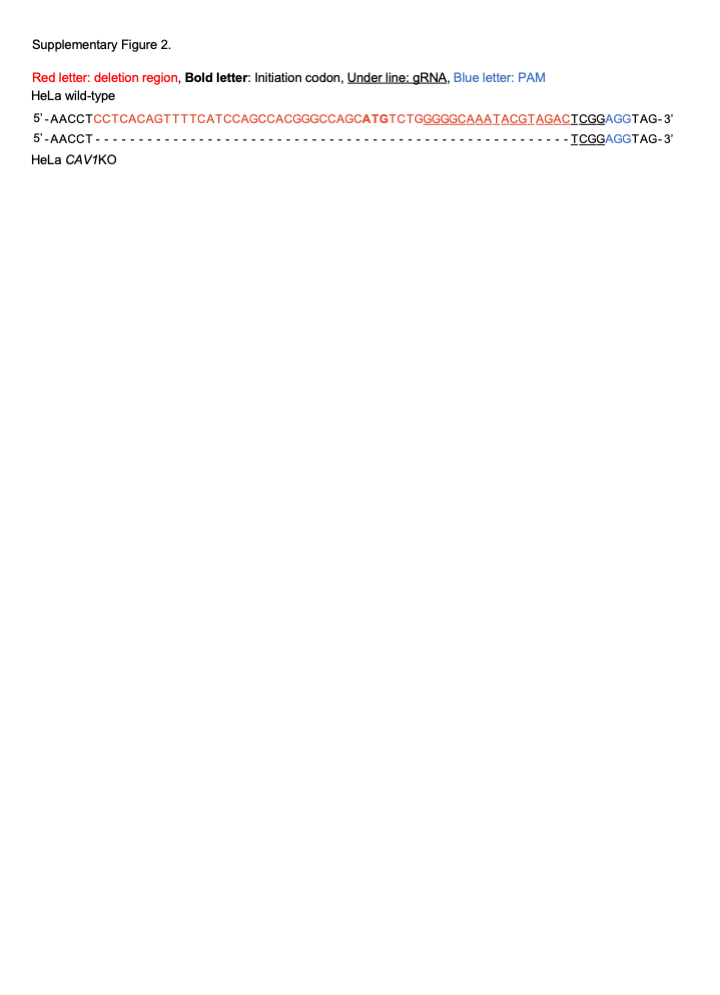

Supplement: Supplementary Figure 2 — Sequence results of genomic CAV1 regions targeted by the gRNA of wild-type and CAV1-KO HeLa cells. Red letters represent the deleted regions, bold letters indicate the initiation codon, underlining shows the gRNA target site, and blue letters represent the PAM motif. [file Image_2.tiff]

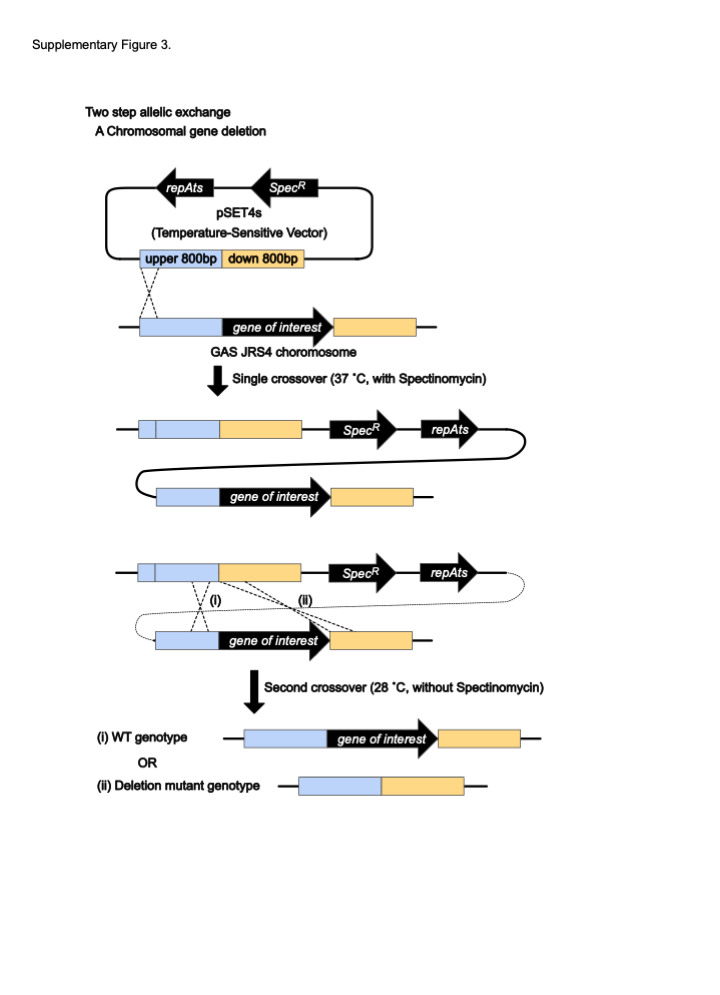

Supplement: Supplementary Figure 3 — Schematic procedure of allelic exchange for (A) chromosomal gene deletion, (B) chromosomal gene complementation, and (C) amino acid substitution in nga. [file Image_3.tiff]
