# Supplementary material for: Group A Streptococcus NAD-Glycohydrolase Inhibits Caveolin 1-Mediated Internalization Into Human Epithelial Cells
Source: Front Cell Infect Microbiol. 2019 Nov 28;9:398. doi: 10.3389/fcimb.2019.00398 (PMC6893971; doi:10.3389/fcimb.2019.00398)

Supplementary Figure 4.

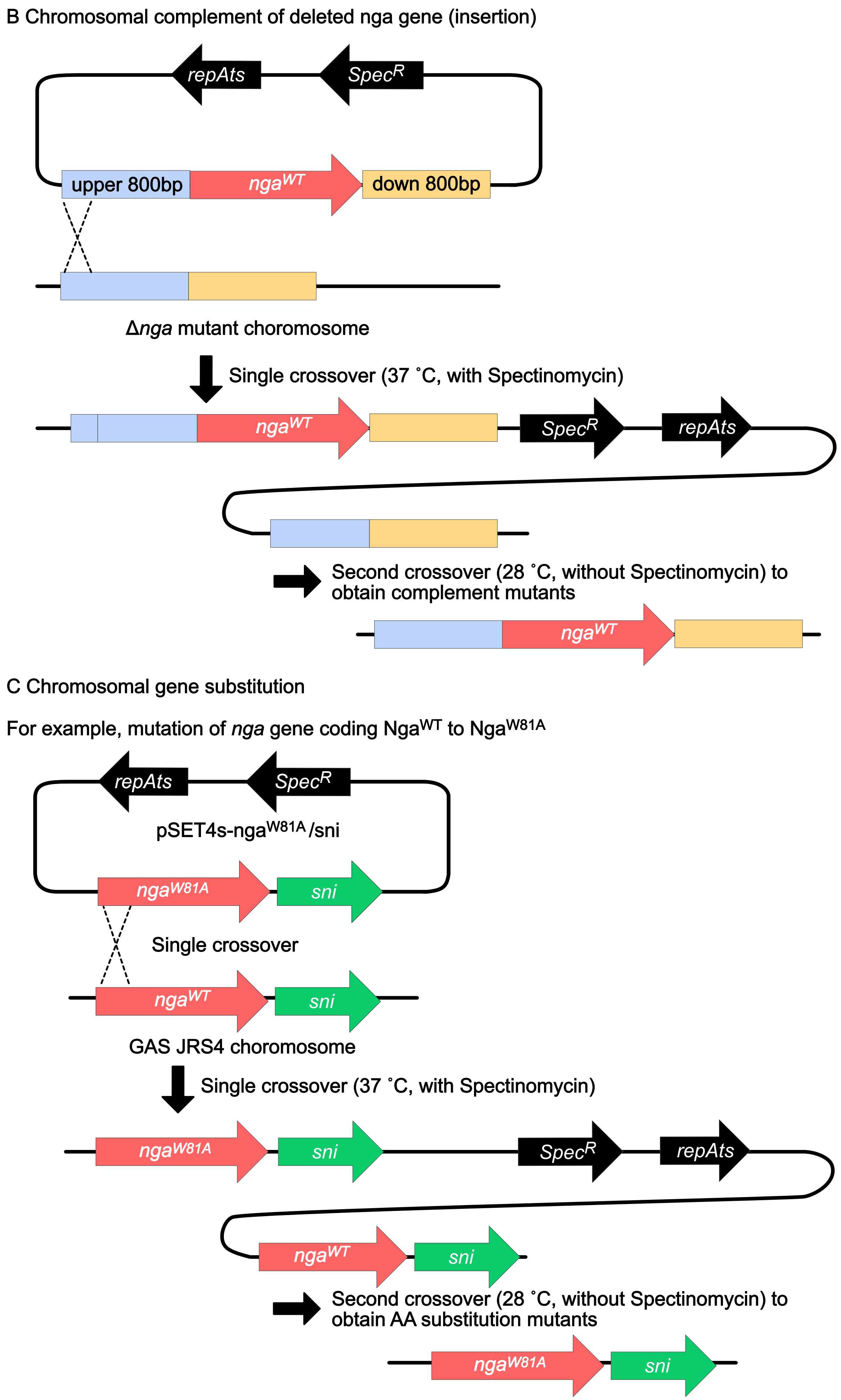

Supplement: Supplementary file 6 [file Image_4.pdf]
